# Synergistic antimicrobial activities of phenolic-rich extract derived from olive pomace and UV-A light against bacterial pathogens and their biofilms

**DOI:** 10.1016/j.crfs.2025.101071

**Published:** 2025-05-09

**Authors:** Yoonbin Kim, Woo-ju Kim, Selina C. Wang, Nitin Nitin

**Affiliations:** a Department of Food Science and Technology, University of California-Davis, Davis, CA, 95616, USA; b Department of Food Science and Biotechnology, Seoul National University of Science and Technology, Seoul, 01811, Republic of Korea; c Research Institute of Food and Biotechnology, Seoul National University of Science and Technology, Seoul, 01811, Republic of Korea; d Department of Biological and Agricultural Engineering, University of California-Davis, Davis, CA, 95616, USA

**Keywords:** Agricultural byproduct, Plant-derived extract, Olive pomace extract, UV-A light, Antimicrobial synergism, Antimicrobial mechanism, Antibiofilm activity

## Abstract

Decontamination of surfaces in food handling and processing environments is a key food safety requirement. In this study, an antimicrobial phenolic-rich aqueous extract derived from olive pomace was combined with UV-A light for the inactivation of bacterial pathogens and their biofilms formed on a plastic surface. The potential antimicrobial synergism between OPE and UV-A light was evaluated against *Escherichia coli* O157:H7 and *Listeria innocua* and quantitatively assessed using isobologram analysis. In addition, the antimicrobial mechanisms and antibiofilm potential of the combined treatment were evaluated. The results demonstrated that OPE and UV-A light exhibited strong synergistic activities (interaction index [*γ*] < 1) and achieved more than a 5-log reduction of planktonic *E. coli* O157:H7 and *L. innocua* cells within 30 min, respectively. Among the major phenolic components of OPE, 4-hydroxyphenylacetic acid (4-HPA) and hydroxytyrosol (HT) exhibited strong synergistic activities with UV-A light. Mechanistic studies revealed that the combined treatment of OPE and UV-A light synergistically induced oxidative stress, membrane damage, and metabolic inhibition in bacterial cells. Furthermore, the combined treatment effectively inactivated bacterial biofilms formed on plastic surfaces and achieved more than a 5-log reduction of *E. coli* O157 and *L. innocua* biofilms within 60 min, respectively. Overall, the findings of this study highlight the strong synergistic potential of diverse phenolic compounds in OPE and UV-A light as an effective intervention strategy. This study provides valuable and practical insights into leveraging antimicrobial extracts derived from agricultural byproducts in combination with mild food processing technologies to enhance the microbial safety of food handling and processing environments.

## Highlights

• An antimicrobial strategy was developed using a plant extract and UV-A light.
• Aqueous olive pomace extract (OPE) was prepared via ultrasound-assisted extraction.
• OPE exhibited strong synergistic antimicrobial activity with UV-A light.
• The synergistic activity was achieved through diverse antimicrobial mechanisms.
• The combined treatment of OPE and UV-A effectively inactivated bacterial biofilms.

## Graphical abstract

**Figure:**
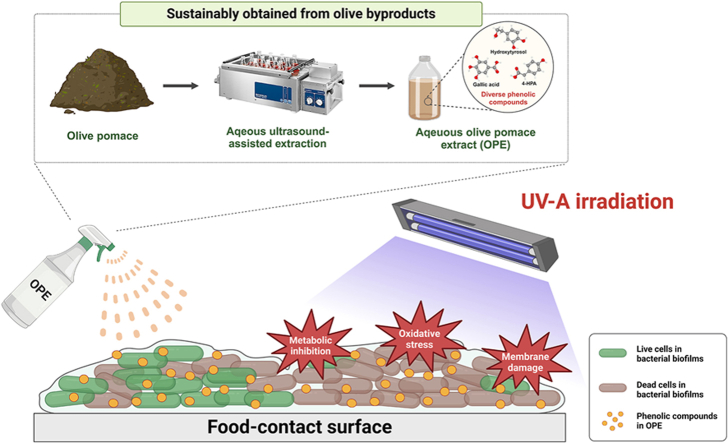

## 1 Introduction

Contamination of foods and food-handling environments with bacterial pathogens is a serious food safety concern. To control the introduction and spread of these pathogens, various chemical sanitizers, such as chlorine-based sanitizers, quaternary ammonium compounds, and peracetic acid have been widely used in the food sector (Cacciatore et al., 2021; Dubey et al., 2022). However, extensive use of these sanitizers is not considered a sustainable approach, as the residues of these sanitizers pose health and environmental risks (Dubey et al., 2022; Musee et al., 2023). For example, the potential formation of toxic halogenated disinfection byproducts (DPBs), such as trihalomethanes (THMs) and haloacetic acid (HAAs), through the reaction between chlorine-based sanitizers and food organic matter limits the broader application of these sanitizers in the food industry (Hung et al., 2017; Shen et al., 2016). This is because certain DBPs, such as trihalomethanes (THMs) and haloacetic acid (HAAs), have been associated with adverse health effects, including potential carcinogenicity and reproductive toxicity (Gil et al., 2009; Hung et al., 2017; Shen et al., 2016). In addition, significantly reduced antimicrobial activities have been reported by these sanitizers against bacterial biofilms formed on food-contact surfaces (Alvarez-Ordóñez et al., 2019; Doh et al., 2022; Galié et al., 2018). It has been reported that bacterial cells embedded in the protective, extracellular polymeric substance (EPS) layer can become up to 1000-fold more resistant to antimicrobial agents than planktonic cells (Davies, 2003; Luppens et al., 2002). Due to their persistence, biofilms formed on food-related surfaces have been considered a leading cause of food contamination, and up to 60 % of all foodborne outbreaks were estimated to be associated with microbial biofilms on food-handling surfaces (Bridier et al., 2015; Liu et al., 2023). Therefore, the development of an alternative approach that addresses both the sustainability concerns of conventional sanitizers and the challenges posed by bacterial biofilms is crucial for ensuring food safety.

Plant-derived antimicrobial extracts have received increasing research attention as promising alternatives to conventional sanitizers (Pinto et al., 2023; Rasheed et al., 2024; Subramani et al., 2017). Plant-derived extracts generally possess low health risks and are environmentally and economically sustainable, as these extracts can be derived from agricultural byproducts (Clewell et al., 2016; Mármol et al., 2021; Qian et al., 2023). Furthermore, compared to conventional sanitizers, plant-derived extracts possess a lower possibility of developing antimicrobial resistance in bacterial pathogens, as they often contain diverse antimicrobial components that can act on different target sites on bacterial cells (Rasheed et al., 2024; Upadhyay et al., 2014). For example, olive pomace, a major byproduct of olive oil production, is rich in phytochemicals known for their antioxidant and antimicrobial activities (Zhao et al., 2022). Diverse phenolic compounds, including gallic acid, hydroxytyrosol, and 4-hydroxyphenyl acetic acid, have been identified in the aqueous extract obtained from olive pomace and exhibited synergistic antimicrobial activities against bacterial pathogens (Kim et al., 2024b; Zhao et al., 2022). However, the broader application of plant-derived extracts in the food industry is still limited. Plant-derived antimicrobials generally require a relatively longer treatment time than the aforementioned conventional sanitizers (Huang et al., 2019; Kim et al., 2024a). Although the overall treatment time can be reduced by using concentrated extracts at the point of application, this may limit the cost-effectiveness of using plant extracts (Li et al., 2021; Ofosu et al., 2020; Sridhar et al., 2021). In addition, the use of concentrated extract might cause handling issues, as increased viscosity may leave undesired residues on target surfaces. For these reasons, plant-derived extracts are often used as natural preservatives for food applications, but not as sanitizing agents that can achieve rapid inactivation of bacterial pathogens present on foods and food-handling surfaces (Efenberger-Szmechtyk et al., 2021; Oulahal and Degraeve, 2022; Shah and Mir, 2022; Tayel et al., 2018).

Ultraviolet (UV) irradiation is recognized as one of the most energy-efficient, non-thermal processing technologies in the food sector (Koutchma, 2019; Tchonkouang et al., 2023). While UV-B (280–320 nm) and UV-C (200–280 nm) lights have been widely used in food applications (Koutchma, 2019; Tchonkouang et al., 2023), UV-A (320–400 nm) light is gaining increasing attention due its unique advantages. The longer wavelengths of UV-A light allow deeper penetration into food matrix with lower energy consumption compared to shorter-wavelength UV lights (Hales and Bastarrachea, 2021; Jeon and Ha, 2020). In addition, unlike UV-B and UV-C lights, UV-A light possesses lower possibilities of inducing photodegradation of nutrients and pigments in foods (Delorme et al., 2020; Falguera et al., 2011; Lante et al., 2016). Although UV-A light itself exhibits low bactericidal activities, it can sensitize the food-grade photoactive compounds, such as phenolic acids and polyphenols, and induce photodynamic inactivation (PDI) of bacterial pathogens through diverse modes of action (de Oliveira et al., 2021; Hales and Bastarrachea, 2021; Jeon and Ha, 2020). In addition, UV-A light can indirectly induce synergistic antimicrobial activities by disrupting bacterial membranes, thereby facilitating the permeation of food-grade antimicrobials into the cytoplasm (Bosshard et al., 2010; de Oliveira et al., 2021; Wang et al., 2017). Despite these promising findings, most previous studies have focused on combining UV-A light with pure phenolic compounds rather than with plant-derived extracts containing diverse phenolic components (de Oliveira et al., 2021; Jeon and Ha, 2020; Park and Kang, 2021; Wang et al., 2017). This represents a knowledge gap in understanding the synergistic antimicrobial potential of phenol-rich, plant-derived extracts and UV-A light. Furthermore, to the best of our knowledge, the combined treatment of food-grade antimicrobials with UV-A light to target bacterial biofilms on food-contact surfaces has not been actively explored.

Previously, we identified over 24 phenolic compounds in the aqueous extract obtained from olive pomace (Kim et al., 2024b). For further evaluating the potential of olive pomace extract (OPE), the current study aims to explore the synergistic antimicrobial potential of the extract and UV-A light against foodborne bacterial pathogens and their biofilms. This study also aims to elucidate antimicrobial mechanisms underlying the synergistic activities between the phenolic components in OPE and UV-A light. To achieve this goal, the antimicrobial synergism between OPE and UV-A light was quantitatively assessed using isobologram analysis, and the phenolic components in OPE contributing to the synergistic activities were identified. In addition, antimicrobial mechanisms underlying the combined treatment of OPE and UV-A light were explored against planktonic bacterial cells. Finally, the synergistic antibiofilm potential of the combined treatment was evaluated. The findings in this study will provide practical insights into developing an effective alternative to conventional sanitizers by synergistically enhancing the antimicrobial efficacy of the plant-derived extracts using a mild food process.

## 2 Materials and methods

### 2.1 Chemicals and reagents

Phosphate-buffered saline (PBS; pH 7.4), tryptic soy agar (TSA), tryptic soy broth (TSB), and SYTOX Orange were purchased from Thermo Fisher Scientific Inc. (Waltham, MA, USA). Folin-Ciocalteu's reagent, sodium carbonate (Na_2_CO_3_), sodium chloride (NaCl), glucose, 1 × M9 medium, tryptone, maximum recovery diluent (MRD), Tween 20, and resazurin sodium salt were purchased from Sigma-Aldrich (St. Louis, MO, USA). Ultrapure water (18 MΩ cm) was obtained using the in-lab Milli-Q RG water ultra-purification system from EMD Millipore (Billerica, MA, USA).

### 2.2 Preparation of olive pomace extract (OPE)

Fresh Arbequina olive pomace was collected from California Olive Ranch (Artois, CA, USA) in 2019 and steam-blanched, pitted, drum-dried, and milled as described by Zhao et al. (2022) to obtain dried olive pomace. Aqueous extracts were obtained from the dried olive pomace using ultrasound-assisted extraction, following the procedures described by Kim et al. (2024b). Briefly, 4 g of dried olive pomace was immersed in 20 mL of deionized water (DW) and bath-sonicated for 30 min (Branson 2510, 100 W, 42 kHz). After bath-sonication, the mixture was centrifuged at 5000×*g* for 10 min. The supernatant was recovered, filtered through a polyethersulfone (PES) filter (pore size: 0.45 μm), and stored at 4 °C until further use. The crude OPE was filtered through a polyethersulfone (PES) syringe filter (pore size: 0.45 μm) and stored at 4 °C until further use.

### 2.3 Total phenolic content (TPC) of OPE

The total phenolic content (TPC) of the OPE was determined using the Folin-Ciocalteu assay (Saucier and Waterhouse, 1999; Singleton and Rossi, 1965), following the modified procedures described by Kim et al. (2024b). Gallic acid solutions (0.625–10 mg/L) were used as the calibration standard, and the TPC was expressed in mg gallic acid equivalents (GAE) per mL of OPE (mg GAE/mL). The TPC of the original extract was ca. 4.2 mg GAE/mL, and the total mass concentration of phenolic components in OPE was ca. 2.12 mg/mL (Kim et al., 2024b). A 0.1 mg GAE/mL was selected as a sublethal concentration of OPE, based on our previous results (Kim et al., 2024b), and used for the synergistic antimicrobial treatment with UV-A light in the following sections.

### 2.4 Preparation of planktonic bacterial cells

A rifampicin-resistant variant of *Escherichia coli* O157:H7 ATCC 700728 and *Listeria innocua* ATCC 33090 (*L*. *innocua* TVS451) were selected as model bacterial strains for Gram-negative and Gram-positive bacteria, respectively. *E. coli* O157:H7 ATCC 700728 is a Shiga toxin-negative strain that lacks *stx1* and *stx2* genes, and *L*. *innocua* TVS451 is commonly used as a surrogate for *Listeria monocytogenes* (Cossu et al., 2017; Moyne et al., 2013). The cryopreserved bacterial stocks were activated in TSB, and stationary phase cultures of *E. coli* O157:H7 or *L. innocua* (ca. 9.3 log CFU/mL) were prepared following the procedures described by Kim et al. (2024b). Each bacterial inoculum was prepared after washing and resuspending the bacterial cultures with 1 × PBS.

### 2.5 Synergistic antimicrobial activities against planktonic cells

The antimicrobial activities of OPE and UV-A light were evaluated against planktonic *E. coli* O157:H7 and *L. innocua* cells, respectively. A 1 mL of OPE (0.1 mg GAE/mL) was dispensed into each well of a 24-well polystyrene plate (Corning, NY, USA). Each bacterial inoculum was diluted 10-fold with DW (ca. 8.3 log CFU/mL), and 10 μL of the bacterial suspensions were inoculated into the wells containing OPE. The well plate was then placed in a light box equipped with four UV-A lamps (320–400 nm; 18 W; Actinic BL, Philips, Holland) and exposed to UV-A light for 0, 5, 15, and 30 min. The distance between the UV-A lamps and the plate was ca. 8 cm, and the average UV-A light intensity was ca. 3.2 ± 0.2 mW/cm^2^, corresponding to a dose of ca. 5.8 ± 0.4 J/cm^2^ after 30 min (de Oliveira et al., 2017). After the treatment, the bacterial suspensions were collected, serially diluted in PBS, and surface-plated on TSA. The TSA plates were incubated at 37 °C for 48 h before counting the colonies. Bacterial cells treated with DW (without OPE or UV-A light) were included as a negative control, and those treated with OPE (without UV-A light) or DW + UV-A light (without OPE) were included as individual treatment controls to validate the synergistic activities of the combined treatment. The theoretical detection limit of the plate count assay was 1.0 log CFU/mL. The treatment time required to achieve a 5-log reduction of the planktonic bacterial cells, determined after 5 h of treatment (Supplementary Fig. 1), was used for the subsequent isobologram analysis and interaction index calculations.

To quantitatively evaluate the synergistic activities between OPE and UV-A light, an isobologram analysis was performed as described by Huu Nguyen et al. (2022) and Kim et al. (2024b) with slight modifications. Briefly, a 5-log isobole was constructed by plotting the treatment times required for a single treatment (OPE or DW + UV-A) to achieve a 5-log reduction of the bacterial population on the x and y-axis, respectively. Then, the treatment time required for the combined treatment of OPE and UV-A light to achieve a 5-log reduction of the bacterial cells was plotted as a single point (▲) on the same plane with the 5-log isobole. It is considered a synergistic, additive, and antagonistic interaction if the plotted point lies below, on, and above the 5-log isobole, respectively. In addition, the interaction index (*γ*) of OPE and UV-A light was also calculated as follows:

$$Interaction\;index\;\left(\gamma \right)=\frac{x}{a}+\frac{x}{b}$$

where *a* and *b* are the treatment time required for a single treatment of OPE or UV-A light to achieve a 5-log reduction of the tested bacteria, and *x* is the treatment time required for the combined treatment of OPE and UV-A light to achieve the same log reduction. It is considered a synergistic, additive, and antagonistic interaction if *γ* < 1, *γ* = 1, and *γ* > 1, respectively.

### 2.6 Synergistic antimicrobial activities of phenolic components of OPE with UV-A light

Synergistic antimicrobial activities of the phenolic components in OPE and UV-A light were evaluated against planktonic *E. coli* O157:H7 and *L. innocua* cells, respectively. Gallic acid (GA), hydroxytyrosol (HT), and 4-hydroxyphenylacetic acid (4-HPA) were selected as three major water-soluble phenolic components of OPE, based on the chemical compositions of OPE reported in the previous study (Kim et al., 2024b). *E. coli* O157:H7 and *L. innocua* suspensions (ca. 6.3 log CFU/mL) were individually supplemented with 0.1 mg GAE/mL of GA, HT, or 4-HPA and exposed to UV-A light every 10 min for until 60 min following the procedures described in section 2.5. The bacterial populations were enumerated using the plate count assay and compared to those treated with OPE + UV-A light. The theoretical detection limit of the plate count assay was 1.0 log CFU/mL.

To better understand the inactivation kinetics of each treatment, the inactivation curves obtained from the plate count assay were further fitted to the Weibull model using the following equation (Buzrul, 2022; Weibull, 1951):

$$N_{t}=N_{0}*e^{{-\left(\frac{t}{\alpha }\right)}^{\beta }}$$

where *t* is the treatment time, *N*_*0*_ is the initial bacterial count at *t* = 0, *N*_*t*_ is the bacterial count at time *t*, *α* is the scale parameter that represents the treatment time required to achieve a significant reduction in the bacterial count, and *β* is the shape parameter that describes the curvature of the inactivation curve. In this study, the scale parameters of the inactivation curves for GA + UV-A, 4-HPA + UV-A, and HT + UV-A treatments were determined and compared to those of OPE + UV-A.

### 2.7 Antimicrobial mechanisms against planktonic bacterial cells

#### 2.7.1 SYTOX orange-based cell permeation test

The effect of the combined treatment of OPE and UV-A light on the membrane permeability was evaluated using SYTOX orange (SO) following the procedures described by (de Oliveira et al., 2017) with slight modifications. SO is a fluorescent dye that can only permeate membrane-compromised cells and fluoresce upon binding to nucleic acids (Biggerstaff et al., 2006). In this assay, a relatively high concentration of *E. coli* O157:H7 cells (ca. 9.3 log CFU/mL) was used to obtain a sufficient fluorescence signal corresponding to the nucleic acid content and to achieve a larger dynamic range for the measurements using SO. Briefly, *E. coli* O157:H7 cells (ca. 9.3 log CFU/mL) were exposed to OPE and UV-A light for 5 min to induce sublethal damage and recovered after centrifuging at 13,000×*g* and 4 °C for 2 min. *E. coli* O157:H7 cells treated with DW were included as a negative control, and those treated with OPE or DW + UV-A were included as individual treatment controls. The bacterial pellets were then washed with ice-cold PBS and resuspended in PBS containing 5 μM of SO. The mixture was incubated at room temperature for 10 min, and the fluorescent intensity was measured using a fluorescence plate reader (SpectraFluor Plus, Tecan Group Ltd., Sunrise, Austria) with excitation/emission wavelengths at 530/580 nm.

#### 2.7.2 Salt-supplemented medium plating (SMP) assay

The SMP assay was performed to evaluate the effect of the combined treatment of OPE and UV-A light on the membrane integrity of bacterial cells following the procedures described by (Kim et al., 2024b). Briefly, *E. coli* O157:H7 cells (ca. 6.3 log CFU/mL) were treated with DW, DW + UV-A, OPE, or OPE + UV-A light for 0, 5, and 10 min. After the treatment, the bacterial cells were serially diluted in PBS, surface-plated both on TSA and TSA-supplemented with 3 % (w/v) NaCl (TSAN), and incubated at 37 °C for 48 h before counting the colonies. Membrane-compromised cells are expected to grow selectively on TSA, but not on TSAN, due to the loss of osmotic tolerance (Espina et al., 2016). The percent populations of membrane-damaged cells were determined as follows:

$$Membrane\;damaged\;cells\;\left(\%\right)=\frac{N_{TSA}-N_{TSAN}}{N_{TSA}}\times 100$$

where *N*_*TSA*_ is the number of *E. coli* O157:H7 colonies formed on TSA, *N*_*TSAN*_ is the number of *E. coli* O157:H7 colonies formed on TSAN, respectively. The theoretical detection limit of the plate count assay was 1.0 log CFU/mL.

#### 2.7.3 Intracellular thiol content measurement

The potential oxidative stress induced by the combined treatment was evaluated by measuring the intracellular thiol content of *E. coli* O157:H7 cells before and after the treatment, following the procedures described by Kim et al. (2024b) with slight modifications. In this assay, a relatively high concentration of *E. coli* O157:H7 cells (ca. 9.3 log CFU/mL) was used to obtain a sufficient thiol content for reliable detection of fluorescence signals. Briefly, *E. coli* O157:H7 cells (ca. 9.3 log CFU/mL) were treated with DW, DW + UV-A, OPE, or OPE + UV-A for 5 min and recovered by centrifuging at 13,000×*g* and 4 °C for 2 min. The bacterial pellets were washed twice with ice-cold PBS, transferred to a Lysing Matrix B tube containing 0.1 mm diameter silica beads (MP Biomedials, Irvine, CA, USA), and homogenized three times using a FastPrep®-24 instrument (MP Biomedials, Irvine, CA, USA) at a speed of 6.5 m/s for 60 s, with 5 min of ice incubation between cycles. The bacterial lysate was recovered after centrifuging at 13,000×*g* and 4 °C for 15 min, and the total thiol content in the lysate was quantified using the Measure-iT™ Thiol Assay Kit (Invitrogen, Waltham, MA, USA) following the manufacturer's instructions. The intracellular content of the bacterial cells treated with DW + UV-A, OPE, and OPE + UV-A was normalized to that of DW-treated cells.

#### 2.7.4 Reactive oxygen species (ROS) quenching assay

The antimicrobial effect of the combined treatment of OPE and UV-A light was evaluated in the presence of thiourea, following the procedures described by de Oliveira et al. (2021) with slight modifications. Thiourea is a well-known scavenger of reactive oxygen species (ROS) generated in biological systems (Foti et al., 2012; Liu and Imlay, 2013). Briefly, *E. coli* O157:H7 cells (ca. 6.3 log CFU/mL) were pre-incubated in DW, with and without 100 mM of thiourea, in the dark at room temperature for 10 min. After incubation, both bacterial suspensions were supplemented with OPE and exposed to UV-A light for up to 30 min. The bacterial cells incubated in thiourea solution for 30 min (without OPE or UV-A light) were included as a control. Treated *E. coli* O157:H7 cells were then serially diluted in PBS, surface-plated on TSA, and incubated at 37 °C for 48 h before counting the colonies. The theoretical detection limit of the plate count assay was 1.0 log CFU/mL.

#### 2.7.5 Inhibition of metabolic activities

The effect of the combined treatment of OPE and UV-A light on the metabolic activities of *E. coli* O157:H7 and *L. innocua* cells was evaluated using a resazurin-based metabolic assay, following the procedures described by Kim et al. (2024b) with minor modifications. Briefly, bacterial cells (ca. 6.3 log CFU/mL) were treated with DW, DW + UV-A, OPE, or OPE + UV-A for 5 min (*E. coli* O157:H7) or 10 min (*L. innocua*) and recovered by centrifuging at 13,000×*g* for 2 min. The bacterial pellets were washed twice with PBS and resuspended in TSB containing 50 μM of resazurin. The mixture was then dispensed into a 96-well polystyrene plate (Corning, NY, USA), and the fluorescence intensity of each sample was monitored during 20 h of incubation at 37 °C using a fluorescence plate reader with excitation/emission wavelengths at 530/580 nm. TSB containing 50 μM of resazurin (without bacterial cells) was used as a blank for each measurement.

### 2.8 Preparation of bacterial biofilms

Four-day-old bacterial biofilms were grown in a 24-well polystyrene plate following the procedures described by Kim et al. (2024a) with slight modifications. Briefly, the *E. coli* O157:H7 or *L. innocua* culture (ca. 9.3 log CFU/mL) was diluted 100-fold in 1 × M9 medium supplemented with 0.4 % glucose and 0.4 % tryptone, and 1 mL of the diluted bacterial suspension (ca. 7.3 log CFU/mL) was dispensed into each well. The well plates were incubated in the dark at room temperature for 4 days. After incubation, the medium was carefully removed, and the bacterial biofilms formed at the bottom of the wells were gently washed twice with 1 mL of PBS to remove loosely attached cells. The washed biofilms were air-dried at room temperature for 30 min and then used in the antibiofilm assays.

### 2.9 Synergistic antibiofilm activities of OPE + UV-A light

The synergistic antibiofilm activities of OPE and UV-A light were evaluated against *E. coli* O157:H7 and *L. innocua* biofilms. A 1 mL of OPE (0.1 mg GAE) was added to each well containing bacterial biofilms prepared as described above (section 2.8). The well plate was then placed in a light box and exposed to UV-A light for up to 60 min. Bacterial biofilms treated with DW were included as a negative control, and those treated with OPE or DW + UV-A were included as individual treatment controls. After treatment, OPE was removed from each well, and 1 mL of MRD supplemented with 1 % (v/v) of Tween 20 was added to each well. The well plate was covered with an adhesive plate seal (Fisher Scientific Inc., MA, USA), bath-sonicated for 2 min, and vortexed at maximum speed for 1 min. The bacterial suspension in each well was serially diluted in PBS, surface-plated on TSA, and incubated at 37 °C for 48 h before counting the colonies. The theoretical detection limit of direct plating was 0.75 log CFU/cm^2^. The treatment time required to achieve a 5-log reduction of bacterial cells in biofilms, determined after 10 h of treatment (Supplementary Fig. 2), was used for the subsequent isobologram analysis and interaction index calculations following the procedures described in section 2.5.

### 2.10 Statistical analysis

Statistical analysis was performed using the GraphPad Prism software V.9.5.1 (Graphpad Software, Inc., La Jolla, CA, USA). All experiments were performed at least in triplicates. The significant differences between treatments were determined through one-way analysis of variance (ANOVA) followed by Tukey's pairwise comparisons with a 95 % confidence interval (*p* < 0.05).

## 3 Results and discussions

### 3.1 Synergistic antimicrobial activities of OPE and UV-A light against planktonic bacterial cells

**Fig. 1:**
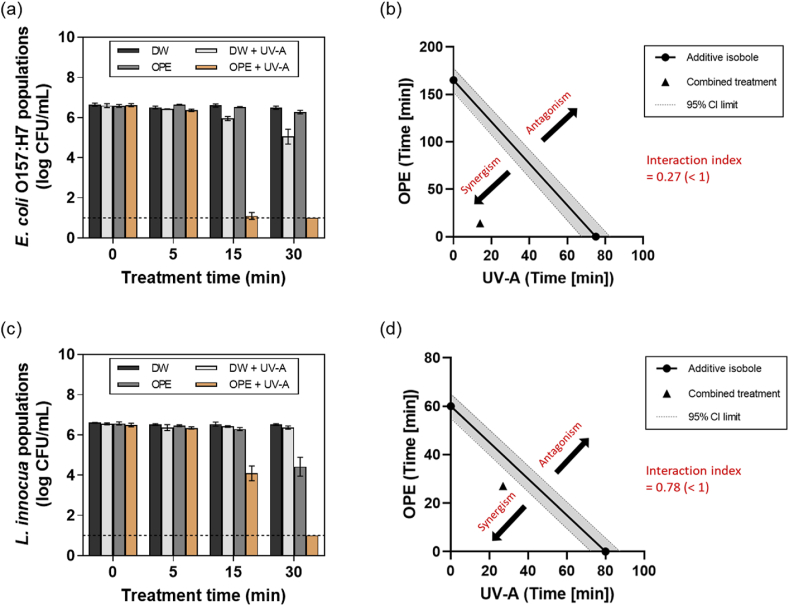
Synergistic antimicrobial activities of OPE and UV-A light against planktonic bacterial cells. Populations of (a) *E. coli* O157:H7 and (c) *L. innocua* cells treated with DW, DW + UV-A, OPE, or OPE + UV-A for up to 30 min. A 5-log isobole was constructed based on the treatment time required for OPE or UV-A alone to achieve a 5-log reduction of (b) *E. coli* O157:H7 and (d) *L. innocua* cells. The treatment time required for the combined treatment of OPE and UV-A light to achieve the same log reduction was plotted as a single point (▲). The results represent the mean values and their standard deviations (*n* = 3), and the theoretical detection limit of direct plating (dashed line) was 1.0 log CFU/mL.

The synergistic antimicrobial activities of the combined treatment of OPE and UV-A light were evaluated against planktonic bacterial cells. Fig. 1a and **c** show the populations of *E. coli* O157:H7 and *L. innocua* cells exposed to DW, DW + UV-A, OPE, or OPE + UV-A for 30 min, respectively. Notably, populations of *E. coli* O157:H7 cells (ca. 6.6 log CFU/mL) showed more than 5 log reductions within 15 min of the combined treatment and decreased below the detection limit (1.0 mg GAE/mL) within 30 min, whereas those treated solely with UV-A or OPE showed ca. 1.5 and 0.3 log reductions after 30 min, respectively. Populations of L. *innocua* cells (ca. 6.5 log CFU/mL) also showed a significant (*p* ≤ 0.05) decrease within 15 min of the combined treatment and decreased below the detection limit within 30 min, whereas those treated solely with UV-A or OPE showed ca. 0.2 and 2.1 log reductions after 30 min, respectively. These results indicate that the antimicrobial activities of OPE were significantly enhanced by the combined treatment with UV-A light.

The synergistic activities between OPE and UV-A light were quantitively evaluated based on the isobologram analysis. Fig. 1b and **d** illustrate the isobologram constructed based on the time required for OPE, UV-A, or OPE + UV-A to achieve a 5-log reduction of *E. coli* O157:H7 and *L. innocua*, respectively. The result show that the combined treatment reduced the treatment time by ca. 11.8 – and 5.4-fold to achieve a 5-log reduction of *E. coli* O157:H7 cells compared to the single treatment of OPE or UV-A light, respectively. In addition, the combined treatment required ca. 2.2- and 3.0-fold less treatment time to achieve a 5-log reduction of *L. innocua* cells compared to OPE and UV-A light, respectively. The interaction index (*γ*) of the combined treatment was determined as 0.27 and 0.78 against *E. coli* O157:H7 and *L. innocua*, respectively, indicating a strong synergism (*γ* < 1) between OPE and UV-A light treatment.

The antimicrobial activities of aqueous OPE have been well-demonstrated in our previous studies (Kim et al., 2024b; Zhao et al., 2023). Strong antibacterial activities were exerted by OPE at concentrations higher than 0.2 mg GAE/mL and achieved more than 5 log reductions of *E. coli* O157:H7 cells within 30 min. These antibacterial activities were attributed to the synergistic interactions between the diverse phenolic components in OPE. In this study, the antimicrobial activities of OPE were further synergized by the UV-A light irradiation. Through the combined treatment with UV-A light, a similar level of bacterial inactivation could be achieved at a lower concentration of OPE (ca. 0.1 mg GAE/mL) within a shorter treatment time (15 min). In addition, it is noteworthy that the concentration of OPE used for the combined treatment corresponds to ca. 0.16 % (w/w) based on the total dry matter of the extract. Given the substantial volumes of sanitizers regularly used in the food industry, the application of a diluted extract in large volumes can be highly advantageous for the routine sanitation of food-handling surfaces (Oulahal and Degraeve, 2022). In addition, it has been estimated that the selling price of phenol-rich extracts (ca. 50 mg GAE/mL) obtained from olive pomace can be as low as $0.82/kg (Orive et al., 2021). This further highlights the economic viability of OPE as a promising alternative to conventional sanitizers. Overall, these findings suggest that the combined treatment of diluted OPE and UV-A light holds significant potential for practical, cost-effective applications in food processing environments.

### 3.2 Synergistic antimicrobial activities of phenolic components in OPE with UV-A light

**Fig. 2:**
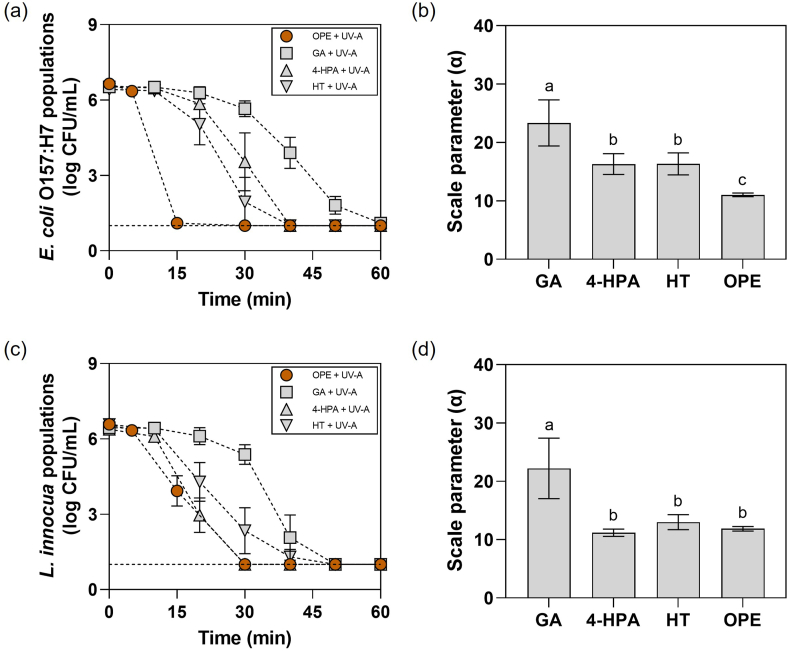
Synergistic antimicrobial activities of phenolic components in OPE (GA, 4-HPA, and HT) with UV-A light. Populations of (a) *E. coli* O157:H7 and (c) *L. innocua* cells treated with OPE + UV-A, GA + UV-A, 4-HPA + UV-A, or HT + UV-A for up to 60 min. Scale parameters (α) of the inactivation curves of (b) *E. coli* O157:H7 and (d) *L. innocua* cells fitted to the Weibull model (R^2^ > 0.99). The theoretical detection limit of direct plating (dashed line) was 1.0 log CFU/mL. The results represent the mean values and their standard deviations (*n* ≥ 3), and different lowercase letters indicate statistically significant differences (*p* < 0.05).

The potential synergistic interaction between phenolic components in OPE and UV-A light was evaluated by fitting the inactivation curves of planktonic bacterial cells to the Weibull model (R^2^ > 0.99). Based on the previous findings, GA, 4-HPA, and HT were selected as three major water-soluble phenolic components in OPE (Kim et al., 2024b). Notably, Fig. 2 illustrates that both 4-HPA and HT exhibited significantly (*p* ≤ 0.05) lower scale parameter (α) than GA when combined with UV-A light against *E. coli* O157:H7 (Fig. 2a and b) and *L. innocua* (Fig. 2c and d) cells, respectively. This indicates that a shorter treatment time was required for 4-HPA or HT with UV-A light to induce a significant decrease in the bacterial populations compared to those of GA with UV-A light. Compared to OPE with UV-A light, 4-HPA and HT showed significantly (*p* ≤ 0.05) higher scale parameters, indicating longer treatment time was required for 4-HPA or HT with UV-A light to induce a significant decrease of *E. coli* O157:H7 populations (Fig. 2b). In contrast, no significant differences (*p* > 0.05) were observed between the scale parameters of the *L. innocua* inactivation curves obtained by 4-HPA, HT, or OPE with UV-A light, indicating that similar treatment time was required for these combined treatments to induce a significant decrease of the *L. innocua* populations (Fig. 2d). However, it is noteworthy that, based on our previous report, the mass fractions of GA, 4-HPA, and HT account for ca. 2.1 %, 16.2 %, and 18.8 % of the total mass of phenolics in OPE, respectively (Kim et al., 2024b). The mass concentration of phenolic compounds in 0.1 mg GAE/mL solutions of GA, 4-HPA, HT, and OPE were ca. 0.1, 0.11, 0.083, and 0.05 mg/mL, respectively (section 2.3). This indicates that the mass concentrations of GA, 4-HPA, and HT in OPE were ca. 90.9-, 13.7-, and 8.83-fold lower than those of the individual phenolic solutions used in this assay. Taken together, our results demonstrate that the diverse phenolic compounds in OPE can synergistically inactivate the bacterial pathogens upon UV-A irradiation. In addition, the results suggest that 4-HPA and HT can exert stronger synergistic activities with UV-A light than GA, and both 4-HPA and HT might have contributed to the strong antimicrobial synergism exhibited by the combined treatment of OPE and UV-A light.

The synergistic antimicrobial properties of the individual phenolic compounds and UV-A light have been extensively studied. For example, de Oliveira et al. (2021) evaluated the potential synergistic antimicrobial activities of different classes of phenolic compounds (cinnamic acid and their derivatives, benzoic acid and their derivatives, and gallic acid and their derivatives) with UV-A light. The authors reported that the tested phenolic compounds exerted significant synergistic activities with UV-A light at a higher concentration (10 mM). However, only cinnamic acid derivatives (ferulic acid, caffeic acid, and coumaric acid) and one benzoic acid derivative (2,5-dihydroxybenzoic acid [DHB]) showed strong synergistic activities at a lower concentration (1 mM). In addition to previous findings, our results suggest that phenolic components of OPE (*e.g.*, GA, 4-HPA, and HT) can exert synergistic activities with UV-A light at varying levels, and these synergistic activities can be further enhanced when multiple phenolic components are simultaneously irradiated with UV-A light in OPE.

### 3.3 Antimicrobial mechanisms of the combined treatment of OPE with UV-A light

#### 3.3.1 Membrane damage

**Fig. 3:**
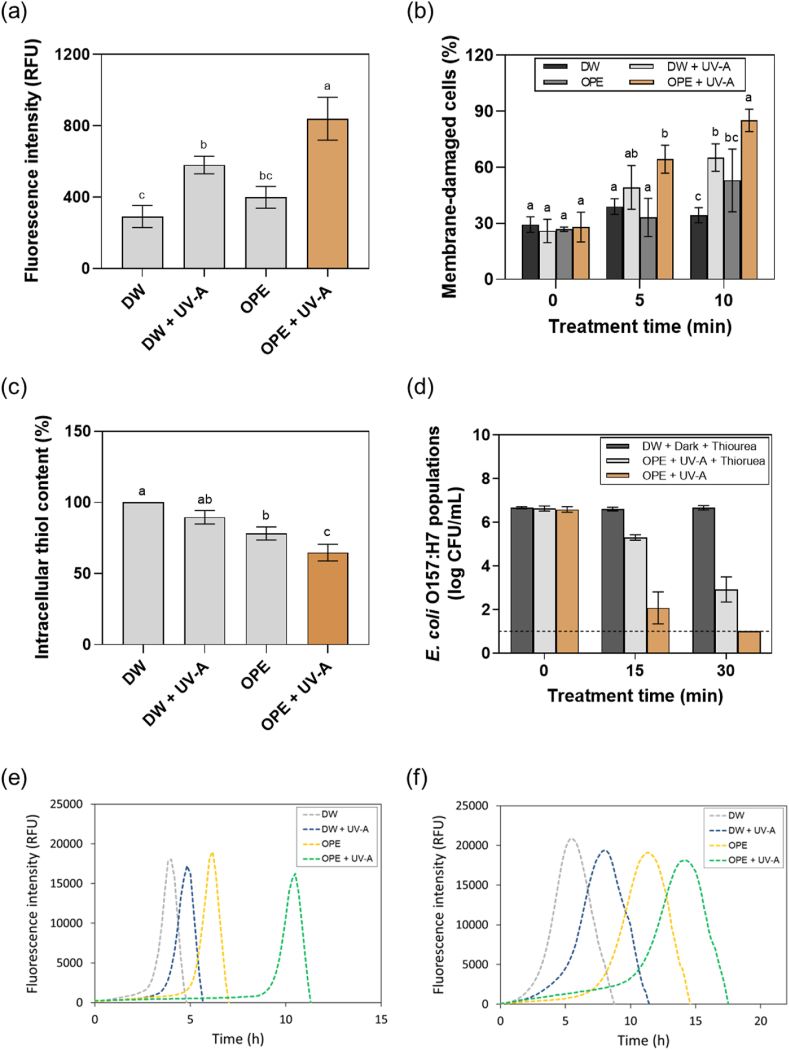
Antimicrobial mechanisms underlying the combined treatment of OPE and UV-A light. The potential membrane damage induced by the combined treatment was evaluated using (a) the SO-based cell permeation test and (b) the SMP assay. The potential oxidative stress generated by the combined treatment was assessed based on (c) intracellular thiol content measurement and (d) the antioxidant addition assay. The inhibitory activities of the combined treatment on the metabolic activities of (e) *E. coli* O157:H7 and (f) *L. innocua* cells were monitored using the resazurin-based metabolic assay. The results represent the mean values and their standard deviations (*n* ≥ 3), and different lowercase letters indicate statistically significant differences (*p* < 0.05).

The potential membrane damage exerted by the combined treatment of OPE with UV-A light was evaluated based on SO membrane permeation and SMP assays, respectively. Fig. 3a illustrates the fluorescence intensity of *E. coli* O157:H7 cells stained with SO after the sublethal treatment (5 min) with DW, DW + UV-A, OPE, or OPE + UV-A. A significant (*p* ≤ 0.05) increase in the fluorescence intensity was observed from the cells treated with OPE + UV-A compared to those treated with DW, DW + UV-A, or OPE. This indicates that bacterial membranes treated with OPE + UV-A become more permeable to SO compared to those treated with DW, DW + UV-A, or OPE. Similar results have been obtained by the SMP assay (Fig. 3b). The results show that an increased number of membrane-damaged *E. coli* O157:H7 cells were observed after the OPE + UV-A treatment compared to DW, DW + UV-A, or OPE, and ca. 85.1 % of the cells were assumed to be membrane-compromised after 10 min of the combined treatment. Taken together, these results indicate that the combined treatment of OPE with UV-A light synergistically disrupted the membrane integrity of the bacterial cells and resulted in increased membrane permeability compared to the individual treatments. It is also noteworthy that an increased level of membrane damage was induced by DW + UV-A compared to DW from both assays. This suggests that UV-A alone may also have contributed to disrupting the bacterial membrane and exerted synergistic activities when combined with OPE.

Synergistic disruptions of bacterial membranes by the combined treatment of pure phenolic compounds and UV-A light have been reported (de Oliveira et al., 2017; Wang et al., 2017). For example, Wang et al. (2017) reported that the combined treatment of GA and UV-A light resulted in a significantly increased level of GA uptake in *E. coli* O157:H7 cells compared to the individual treatment (GA or UV-A). In addition, significantly increased membrane permeability to propidium iodide (PI) dye was observed in *E. coli* O157:H7 cells treated with GA + UV-A compared to those treated with GA or UV-A. The authors described that UV-A light may have compromised the bacterial membrane structures, facilitating the partitioning of phenolic compounds, which could have further disrupted the membrane permeability. Previously, we found that phenolic components in OPE can synergistically disrupt the bacterial membranes, resulting in ca. 93.5 % of *E. coli* O157:H7 cell membranes compromised after 30 min treatment (Kim et al., 2024b). However, in the present study, a similar level of membrane disruption could be achieved within 10 min in the presence of UV-A light at an equivalent OPE concentration (0.1 mg GAE/mL). To the best of our knowledge, this is the first study reporting the synergistic disruption of bacterial membranes by the combination of phenolic-rich, plant-derived extracts and UV-A light. Further studies are needed to investigate the potential biochemical interactions between phenolic components in OPE and bacterial membranes, as well as the role of UV-A light in enhancing these interactions.

#### 3.3.2 Oxidative stress

The potential oxidative stress exerted by the combined treatment of OPE and UV-A light was evaluated based on the intracellular thiol content measurement and ROS quenching assay, respectively. Fig. 3c illustrates the intracellular thiol content of *E. coli* O157:H7 cells measured after 5 min of sublethal treatment with DW, DW + UV-A, OPE, and OPE + UV-A, respectively. Notably, *E. coli* O157:H7 cells treated with OPE + UV-A showed significantly (*p* ≤ 0.05) lower thiol content compared to those treated with DW + UV-A or OPE. The results show that ca. 64.4 % of thiol content remained after the combined treatment whereas ca. 90.0 % and 78.2 % remained after treatment with DW + UV-A and OPE, respectively. This indicates that the combined treatment of OPE with UV-A light exerted a significantly higher degree of oxidative stress inside the cells compared to the individual treatments. Interestingly, a significant decrease of the intracellular content was observed from the cells solely treated with OPE compared to those treated with DW. This indicates that OPE alone might have induced a minor degree of oxidative stress inside the cells, which was synergistically enhanced when combined with UV-A light. The potential oxidative stress induced by the combined treatment was further assessed by supplementing the ROS quencher during the combined treatment (Fig. 3d). The results show that the presence of thiourea significantly attenuated the inactivation of *E. coli* O157:H7 cells during the OPE + UV-A treatment. For example, ca. 2.9 log CFU/ml of *E. coli* O157:H7 cells were enumerated after 30 min of the combined treatment in the presence of thiourea, whereas no cells were enumerated above the detection limit (1.0 log CFU/mL) in its absence. The result indicates that significant oxidative stress was induced on the bacterial cells by the combined treatment and exogenously quenched by the addition of thiourea. Collectively, these results suggest that the combined treatment of OPE and UV-A light may have synergistically induced both intracellular and extracellular oxidative stresses on the bacterial cells, and such oxidative stress might have contributed to the synergistic bactericidal activities exerted by the combined treatment.

The oxidative damage induced by the combined treatment of pure phenolic compounds and UV-A light has been documented by other studies. For example, Wang et al. (2017) reported that significantly higher levels of ROS were intracellularly generated by the combined treatment of GA (15 mM) and UV-A compared to the single GA or UV-A treatment. The authors suggested that ROS may be directly generated by the oxidation of GA in bacterial cells upon UV-A light irradiation or indirectly by activating other intracellular metabolic pathways that lead to ROS accumulation (*e.g.*, self-destructive stress response [Zhao et al., 2015]). In addition, de Oliveira et al. (2021) reported that the addition of ROS-scavengers (i.e., glutathione and thiourea) significantly attenuated the synergistic antimicrobial activities of phenolic compounds and their derivatives (GA, propyl gallate [PG], and DHB) with UV-A light. The authors described that phenolic compounds and their derivatives, such as GA and PG, can generate localized ROS upon exposure to UV-A light, leading to the oxidation of thiol-containing biomolecules within the cells and/or the peroxidation of lipids in the bacterial membrane (de Oliveira et al., 2017, 2021). In the previous study, the intracellular thiol content of *E. coli* O157:H7 cells significantly decreased after 30 min of incubation in OPE (0.1 mg GAE/mL) compared to those treated with DW, GA, HT, or 4-HPA. However, in the present study, a significant decrease of intracellular thiol content could be observed even within 10 min upon UV-A irradiation of OPE.

It is important to note that many of the phenolic compounds in OPE can function as both pro- and antioxidants, depending on certain factors such as concentration, pH, or the presence of metal ions (Aruoma et al., 1993; Dintcheva et al., 2017; Kessler et al., 2003; Khan et al., 2000). For example, Dintcheva et al. (2017) demonstrated that ferulic acid and vanillic acid can exert antioxidant properties at lower concentrations but also can exert prooxidant properties at concentrations higher than a certain threshold. In this regard, it is possible that the oxidative stress exerted by the combined treatment of OPE and UV-A light may reflect the cumulative effects of both pro- and antioxidant activities of the phenolic compounds in OPE. Therefore, further studies are needed to better understand the pro- and antioxidant behaviors of the phenolic components in OPE and to enhance their contribution to the overall antimicrobial effectiveness of the combined treatment.

#### 3.3.3 Inhibition of metabolic activities

The potential inhibitory effects of the combined treatment on the metabolic activities of the bacterial cells were evaluated using resazurin as a fluorescent indicator. Fig. 3e and **f** shows the change in fluorescence intensity of resazurin molecules as they are reduced to resorufin by metabolically active *E. coli* O157:H7 and *L. innocua* cells, respectively, after sublethal treatment with DW, DW + UV-A, OPE, or OPE + UV-A. Notably, both bacterial cells treated with OPE + UV-A required significantly longer incubation time to reach the maximum fluorescent intensity compared to those treated with DW, DW + UV-A, or OPE. For example, ca. 10.5 h was required for *E. coli* O157:H7 cells to reach the maximum fluorescent intensity after OPE + UV-A treatment, whereas ca. 4.0, 4.8, and 6.2 h were required after treatment with DW, DW + UV-A, and OPE, respectively (Fig. 3e). This indicates that the combined treatment of OPE and UV-A light induced a significantly higher degree of damage to the metabolic machinery of the bacterial cells compared to the individual treatments. Such damage was reflected in the extended incubation time required for the bacterial cells to recover their metabolic activities and reach maximum fluorescence intensity (de Oliveira et al., 2017). It is also worth noting that the DW + UV-A or OPE treatment resulted in a delay for the bacterial cells to reach the maximum fluorescence intensity compared to those treated with DW. This indicates that DW + UV-A or OPE treatment induced a minor degree of damage to the metabolic activities of the bacterial cells, and their individual activities were synergistically enhanced when applied together.

Previously, the effect of the combined treatment of GA, lactic acid (LA), and UV-A light on metabolic activities of the bacterial cells has been demonstrated by de Oliveira et al. (2017). The authors reported that a longer delay (ca. 1.0–3.6 h) in reaching the maximum fluorescence intensity was observed from *E. coli* O157:H7 cells treated with GA, LA, or GA + LA upon UV-A irradiation. In addition, a longer delay was observed from the bacterial cells treated with GA + LA + UV-A compared to those treated with GA + UV-A or LA + UV-A. These results indicate that a higher degree of damage was induced on the metabolic activities of the bacterial cells when GA and LA were applied simultaneously with UV-A light. In comparison, a considerably longer incubation time (ca. 10.5 h) was required for *E. coli* O157:H7 cells to reach the peak fluorescence intensity after treatment with OPE (0.1 mg GAE/mL) + UV-A than those treated with GA (ca. 0.17 mg GAE/mL) + UV-A (ca. 6.0 h) (de Oliveira et al., 2017). This further supports that the multiple phenolic components in OPE can be simultaneously photoactivated upon UV-A irradiation, exerting stronger inhibitory activities on the metabolic activities of the bacterial cells even at a lower total phenolic content.

Taken together, the mechanistic evaluations of the combined treatment demonstrated that phenolic components in OPE can synergistically disrupt the membrane integrity, redox balance, and metabolic activities of the bacterial cells upon UV-A light irradiation. However, further holistic approaches such as transcriptomic and metabolomic analyses are needed to understand the antimicrobial mechanisms underlying the combined treatment of different phenolic compounds in OPE and UV-A light. For example, de Oliveira et al. (2021) reported differences in the mode of synergistic antimicrobial activities between cinnamic acid derivatives and benzoic acid derivatives. The authors reported that, unlike other benzoic acid derivatives, cinnamic acid derivatives such as ferulic acid exhibited strong synergistic activities even in the presence of ROS scavengers such as glutathione and thiourea. This indicates that the synergistic antimicrobial activity of ferulic acid and UV-A light may be less dependent on direct ROS generation. Considering the multi-component nature of OPE, different phenolic components may have complementarily interacted with each other and with UV-A light through multiple modes of action, thereby exhibiting strong synergistic activity.

### 3.4 Synergistic antibiofilm activities of OPE with UV-A light

**Fig. 4:**
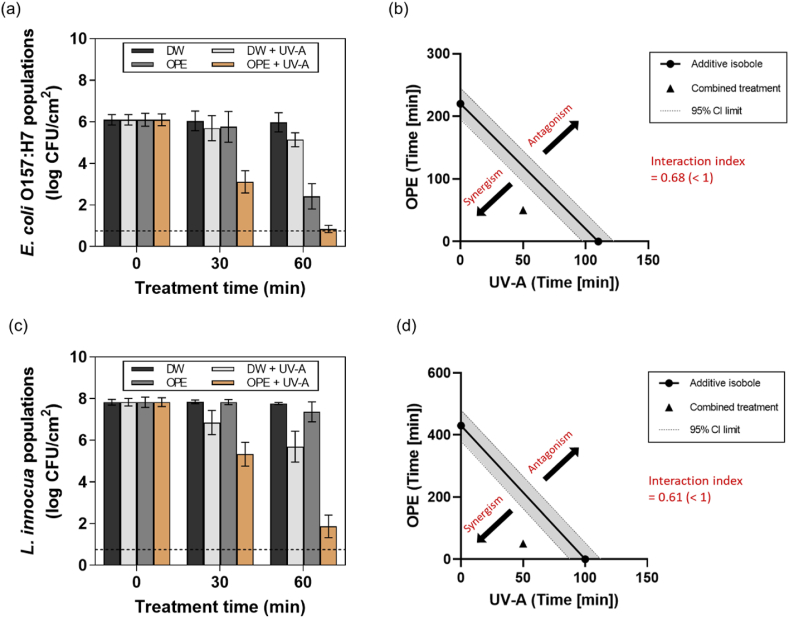
Synergistic antibiofilm activities of OPE and UV-A light. Biofilms of (a) *E. coli* O157:H7 and (c) *L. innocua* were treated with DW, DW + UV-A, OPE, or OPE + UV-A for up to 60 min. A 5-log isobole was constructed based on the treatment time required for OPE or UV-A alone to achieve a 5-log reduction of (b) *E. coli* O157:H7 and (d) *L. innocua* cells in biofilms. The treatment time required for the combined treatment of OPE and UV-A light to achieve the same log reduction was plotted as a single point (▲). The results represent the mean values and their standard deviations (*n* = 3), and the theoretical detection limit of direct plating (dashed line) was 0.75 log CFU/cm^2^.

The synergistic antibiofilm activities of the combined treatment of OPE and UV-A light were evaluated. Fig. 4a and **c** show the populations of the bacterial cells in biofilm after 0, 30, or 60 min of treatment with DW, DW + UV-A, OPE, or OPE + UV-A, respectively. Overall, strong synergistic activities were exerted by the combined treatment of OPE and UV-A light against both bacterial biofilms. Populations of *E. coli* O157:H7 cells in biofilm (ca. 6.1 log CFU/cm^2^) showed ca. 2.9 log reductions within 30 min of the combined treatment and decreased to ca. 0.85 log CFU/cm^2^ (2 out of 3 replicates decreased below the detection limit) after 60 min, achieving more than 6 log reductions. In contrast, *E. coli* O157:H7 cells in biofilms treated with DW, DW + UV-A, or OPE decreased to 6.0, 5.1, or 2.4 log CFU/cm^2^ after 60 min, respectively (Fig. 4a). Similarly, the populations of *L. innocua* cells in biofilm (ca. 7.8 log CFU/cm^2^) showed ca. 2.5 log reductions within 30 min of the combined treatment and achieved ca. 5.9 log reductions after 60 min. However, *L. innocua* cells in biofilms treated with DW, DW + UV-A, or OPE only decreased to ca. 7.8, 5.7, or 7.4 log CFU/cm^2^ after 60 min, respectively (Fig. 4c). These results indicate that strong synergistic activities were exerted by the combined treatment of OPE and UV-A light against the bacterial biofilms. The antibiofilm activities of conventional sanitizers, such as a sodium hypochlorite solution, have been actively investigated. For example, Vaid et al. (2010) reported that *L. monocytogenes* biofilms treated with 50 ppm of sodium hypochlorite solution resulted in ca. 3.1 log reductions after 10 min. Huang et al. (2019) reported that *E. coli* O157:H7 and *L. innocua* biofilms treated with 20 ppm of sodium hypochlorite solution resulted in ca. 2.3 and 1.9 log reductions after 20 min, respectively, and no further reduction was observed after 60 min. In comparison, OPE and UV-A-combined treatment can be a promising alternative to conventional sanitizers for inactivating bacterial biofilms on food-handling surfaces.

The synergistic antibiofilm activities of the combined treatment of OPE and UV-A light were further quantitatively evaluated using the isobologram analysis. Fig. 4b and **d** illustrate the isobologram constructed for DW + UV-A, OPE, or OPE + UV-A treatments to achieve 5 log reductions of the bacterial cells in biofilms. Fig. 4b shows that ca. 2.2 and 4.4-fold shorter treatment time was required for OPE + UV-A to achieve 5 log reductions of *E. coli* O157:H7 cells in biofilm compared to DW + UV-A or OPE treatment, respectively. In addition, ca. 2.0 and 8.6-fold shorter treatment time was required for the combined treatment to achieve 5 log reductions of *L. innocua* cells in biofilm compared to DW + UV-A or OPE treatment, respectively (Fig. 4d). The interaction index (*γ*) of the combined treatment was determined as 0.68 and 0.61 against *E. coli* O157:H7 and *L. innocua* biofilms, respectively, indicating that strong synergistic activities (*γ* < 1*)* were exerted by the combined treatment of OPE and UV-A light compared to the individual treatment.

Although the antimicrobial activities of olive byproduct-derived extracts (*e.g.*, olive leaf extract, olive mill wastewater [OMW], and olive pulp extract) have been widely investigated (Gullón et al., 2018; Thielmann et al., 2017), only limited studies have evaluated the antibiofilm potential of these extracts. Carraro et al. (2014) reported that the addition of 4 mg/mL of OMW significantly (*p* ≤ 0.05) reduced the biofilm formation of *E. coli* K-12 on polystyrene surfaces. The subsequent transcriptomic analysis revealed that the expression of genes involved in biofilm formation and regulation (*bhsA, csgC, rcsA, bssS, bssR, ydaM, yddV, yhjH*) was significantly influenced by sublethal treatment with OMW (1.0 mg/mL). In addition, notable repression of motility and chemotaxis-related genes was observed in *E. coli* K-12 treated with OMW, suggesting that surface colonization and dissemination of bacterial cells may have been inhibited. While the results highlight the potential of the olive byproduct-derived extract in inhibiting bacterial biofilm formation, its efficacy against the biofilms already established on food-related surfaces has not been demonstrated. It is well-known that bacterial cells in biofilms can become considerably more resistant to antimicrobial treatments due to the presence of a protective extracellular polymeric substance (EPS) layer (Alvarez-Ordóñez et al., 2019; Kim et al., 2018, 2019). Previously, Cossu et al., 2016 reported that 1 h of combined treatment of GA (10 mM; ca. 1.7 mg GAE/mL) and UV-A light resulted in ca. 80 % decrease in metabolic activities of *E. coli* O157:H7 cells in biofilms. However, only minor inhibitory activities were exerted when a lower concentration of GA (5 mM; ca. 0.85 mg GAE/mL) was used for the combined treatment. However, in the present study, strong antibiofilm activities were exerted at a significantly lower total phenolic content (ca. 0.1 mg GAE/mL), attributable to the diverse phenolic components of OPE that can exhibit synergistic activities with UV-A light through various modes of action.

Overall, the findings of this study provide useful insights into developing a green antimicrobial treatment using aqueous extracts derived from agricultural byproducts and synergistic strategies to enhance their activity using mild processes such as UV-A light irradiation. The strong synergistic antimicrobial activities of OPE and UV-A light offer a safer alternative to conventional synthetic sanitizers for combating pathogenic bacteria and their biofilms in food-handling environments. In future studies, the potential to improve the inactivation kinetics of OPE + UV-A treatment by using a more concentrated OPE and a higher-intensity UV-A source needs to be evaluated. In addition, feasibility of integrating other mild processing technologies, such as ultrasound or mild heat, to further enhance the antimicrobial activities of the OPE + UV-A treatment needs to be investigated. In this study, significantly enhanced synergistic activities were demonstrated by OPE + UV-A compared to those of individual major phenolic components (GA, 4-HPA, or HT + UV-A). However, future studies may focus on complementary bioanalytical approaches, such as transcriptomic and metabolomic analyses, to gain a deeper understanding of the antimicrobial mechanisms underlying this synergistic treatment. These analyses would also enable the elucidation of antimicrobial interactions between the diverse phenolic components in OPE, including those present at relatively lower concentrations, thereby facilitating the optimization and broader application of this combined antimicrobial strategy.

## 4 Conclusions

In this study, a novel synergistic antimicrobial strategy was developed using the combined treatment of an olive byproduct-derived extract and UV-A light. Aqueous olive pomace extract (OPE) was obtained through a water-based, ultrasound-assisted extraction. UV-A light irradiation of OPE exhibited strong antimicrobial synergisms (interaction index [*γ*] < 1) against planktonic bacterial cells and achieved more than a 5-log reduction of *E. coli* O157:H7 (*γ*: 0.27) and *L. innocua* (*γ*: 0.78) within 30 min, respectively. Among the major phenolics of OPE, 4-hydroxyphenylacetic acid (4-HPA) and hydroxytyrosol (HT) exhibited strong synergistic activities with UV-A light, potentially contributing to the strong antimicrobial synergism exerted by OPE and UV-A light. Antimicrobial mechanistic studies revealed that OPE and UV-A light synergistically disrupted bacterial redox balance, membrane integrity, and metabolic activities. In addition, the combined treatment exerted strong synergistic activities against bacterial biofilms formed on plastic surfaces and achieved more than a 5-log reduction of *E. coli* O157:H7 (*γ*: 0.68) and *L. innocua* (*γ*: 0.61) biofilms within 60 min, respectively. This synergistic approach effectively enhanced the antimicrobial potential of diverse phenolic compounds in OPE and UV-A light through multiple antimicrobial mechanisms. The findings in this study suggest the potential of the OPE and UV-A light combined treatment as an effective alternative to conventional chemical sanitizers.

## CRediT authorship contribution statement

**Yoonbin Kim:** Conceptualization, Methodology, Validation, Formal analysis, Investigation, Writing – original draft, Writing – review & editing, Visualization, Project administration. **Woo-ju Kim:** Methodology, Investigation. **Selina C. Wang:** Conceptualization, Resources, Supervision, Writing – review & editing, Funding acquisition. **Nitin Nitin:** Conceptualization, Methodology, Resources, Supervision, Writing – review & editing, Funding acquisition, Project administration.

## Funding

This research was supported by the 10.13039/100006759California Department of Food and Agriculture (CDFA) 2020 Specialty Crop Block Grant Program (20-0001-033-SF) and the 10.13039/100000199U.S. Department of Agriculture 10.13039/100005825National Institute of Food and Agriculture (USDA-10.13039/100005825NIFA) Foundational Food Safety Program (Grant No. 2022-67017-36308).

## Declaration of competing interest

The authors declare that they have no known competing financial interests or personal relationships that could have appeared to influence the work reported in this paper.

## Data Availability

Data will be made available on request.
