# Supplementary material for: Synergistic antimicrobial activities of phenolic-rich extract derived from olive pomace and UV-A light against bacterial pathogens and their biofilms
Source: Curr Res Food Sci. 2025 May 9;10:101071. doi: 10.1016/j.crfs.2025.101071 (PMC12136893; doi:10.1016/j.crfs.2025.101071)
Supplement: Multimedia component 1 [file mmc1.docx]

**Supplementary Information**

**Synergistic antimicrobial activities of phenolic-rich extract derived from olive pomace and UV-A light against bacterial pathogens and their biofilms**

Yoonbin Kim^a^, Woo-ju Kim^b,c^, Selina C. Wang^a^ and Nitin Nitin^a,d,^*

^a^Department of Food Science and Technology, University of California-Davis, Davis, CA 95616, USA

^b^Department of Food Science and Biotechnology, Seoul National University of Science and Technology, Seoul, 01811, Republic of Korea

^c^Research Institute of Food and Biotechnology, Seoul National University of Science and Technology, Seoul, 01811, Republic of Korea

^d^Department of Biological and Agricultural Engineering, University of California-Davis, Davis, CA 95616, USA

* Corresponding author.

Correspondence to Dr. Nitin Nitin
Mailing address: 2214 Robert Mondavi Institute - South building, 1 Shields Ave, Davis, CA, 95616
Telephone: (530) 752-6208, Fax: (530) 752-4759
E-mail address: nnitin@ucdavis.edu

***Inactivation kinetics of planktonic bacterial cells***

The inactivation kinetics of planktonic *E. coli* O157:H7 and *L. innocua* cells treated with OPE, DW + UV-A, or OPE + UV-A were measured following the procedures described in section 2.5 with extended treatment time (for up to 5 h). The treatment time required for OPE, DW + UV-A, or OPE + UV-A to achieve 5-log reductions of planktonic *E. coli* O157:H7 and *L. innocua* cells were determined as ca. 2.75, 1.25, and 0.23 h and ca. 1.0, 1.33, and 0.45 h, respectively (**Supplementary Figure 1**). These 5-log reduction times were used in the subsequent isobologram analysis and interaction index calculation described in section 2.5.

***Inactivation kinetics of bacterial cells in biofilms***

The inactivation kinetics of *E. coli* O157:H7 and *L. innocua* cells in biofilms treated with OPE, DW + UV-A, or OPE + UV-A were measured following the procedures described in section 2.9 with extended treatment time (for up to 10 h). The treatment time required for OPE, DW + UV-A, or OPE + UV-A to achieve 5-log reductions of *E. coli* O157:H7 and *L. innocua* cells in biofilms were determined as ca. 3.67, 1.83, and 0.83 h and ca. 7.17, 1.67, and 0.83 h, respectively (**Supplementary Figure 2**). These 5-log reduction times were used in the subsequent isobologram analysis and interaction index calculation described in section 2.9.

**
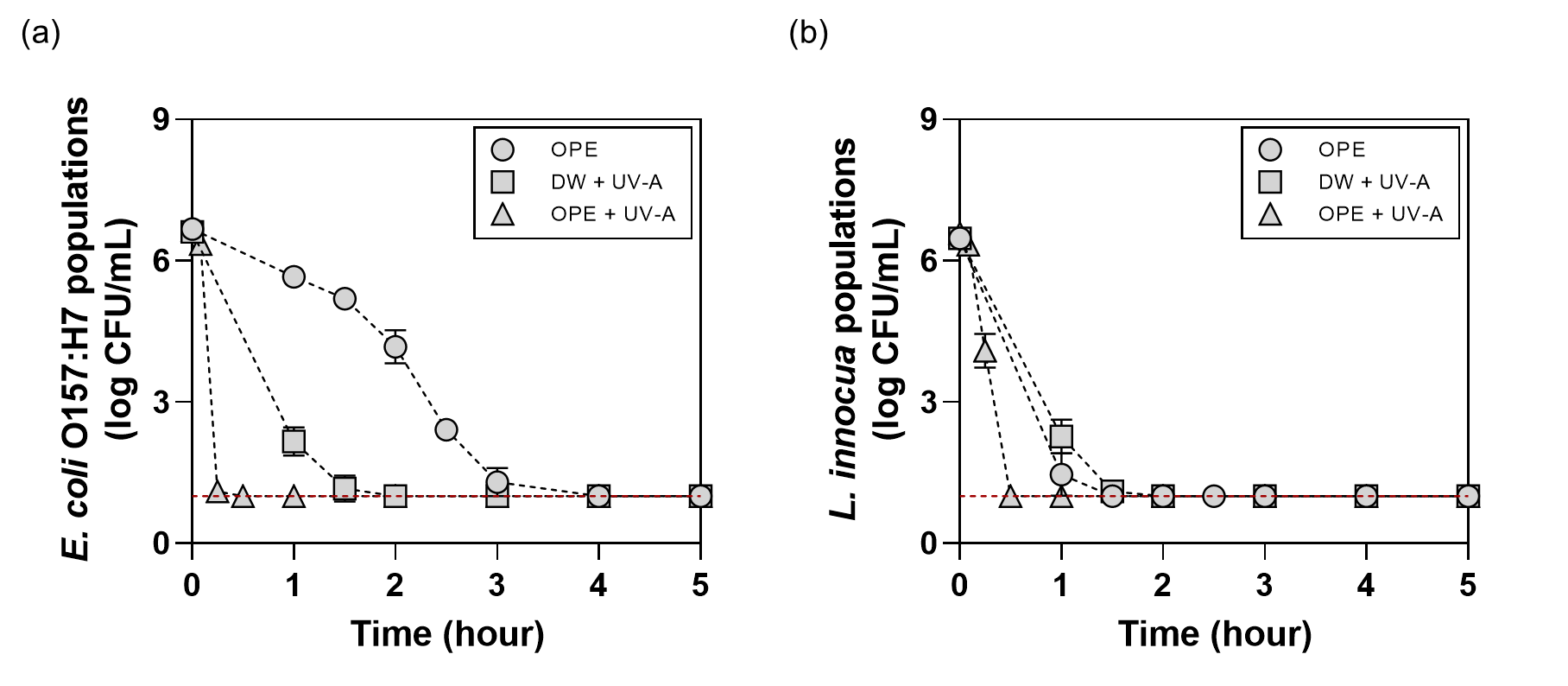
**

**Supplementary Figure 1.** Inactivation kinetics of planktonic (a) *E. coli* O157:H7 and (b) *L. innocua* cells treated with OPE, DW + UV-A, or OPE + UV-A at room temperature for up to 5 h. After treatment, bacterial cells were enumerated using a plate count assay, and the theoretical detection limit of direct plating (red dashed line) was 1.0 log CFU/cm^2^. The results represent the mean values and their standard deviations (*n* = 3).

**
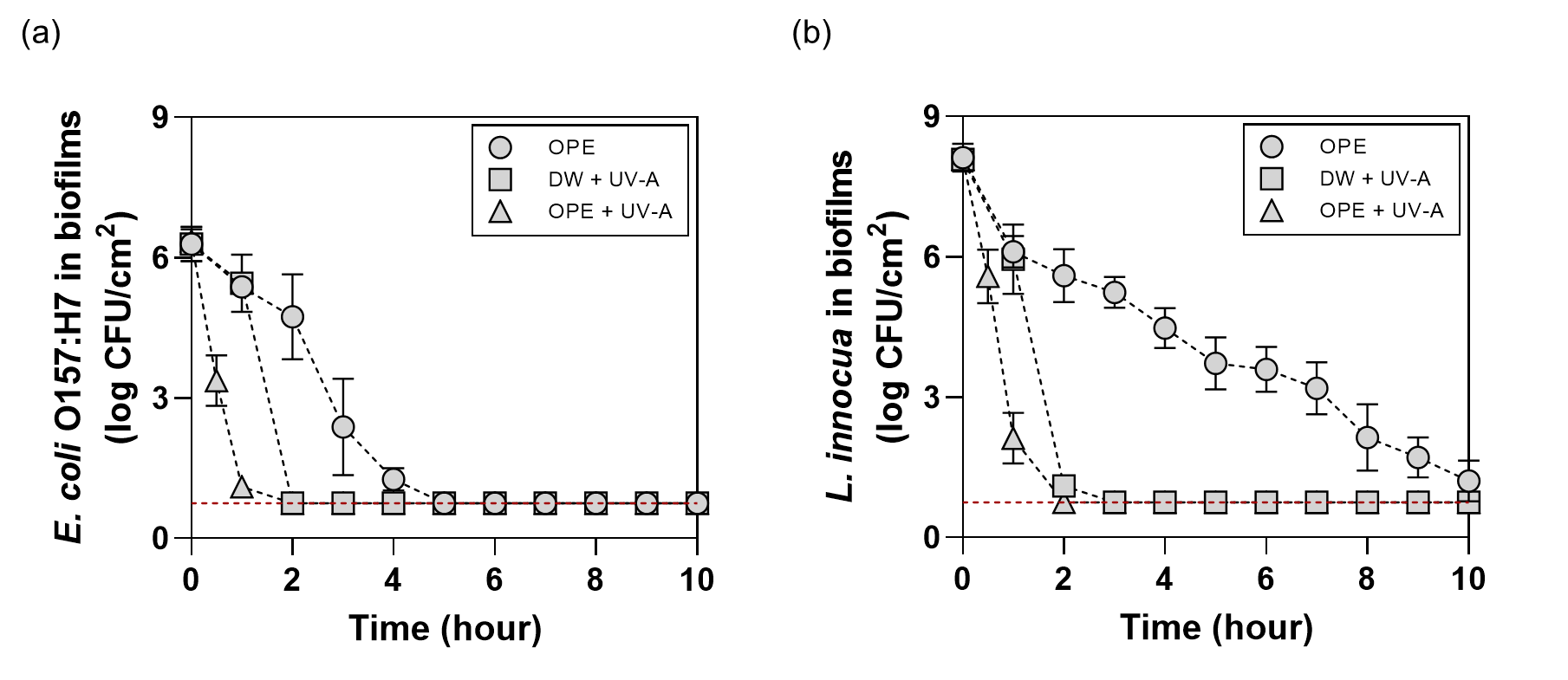
**

**Supplementary Figure 2.** Inactivation kinetics of (a) *E. coli* O157:H7 and (b) *L. innocua* cells in biofilms treated with OPE, DW + UV-A, or OPE + UV-A at room temperature for up to 10 h. After treatment, bacterial cells were recovered using MRD supplemented with 1% (v/v) Tween 20 and enumerated using a plate count assay. The theoretical detection limit of direct plating (red dashed line) was 0.75 log CFU/cm^2^, and the results represent the mean values and their standard deviations (*n* = 3).
